# Applied Bayesian Approaches for Research in Motor Neuron Disease

**DOI:** 10.3389/fneur.2022.796777

**Published:** 2022-03-24

**Authors:** Anna G. M. Temp, Marcel Naumann, Andreas Hermann, Hannes Glaß

**Affiliations:** ^1^Translational Neurodegeneration Section “Albrecht Kossel,” Department of Neurology, University Medical Centre, Rostock, Germany; ^2^Deutsches Zentrum für Neurodegenerative Erkrankungen (DZNE), Rostock, Germany; ^3^Neurozentrum, Berufsgenossenschaftliches Klinikum Hamburg, Hamburg, Germany; ^4^Center for Transdisciplinary Neurosciences Rostock, University Medical Centre, Rostock, Germany

**Keywords:** Bayesian statistics, amyotrophic lateral sclerosis, single case studies, clinical trials, tofersen, JASP, synthetic data, motor neurone disease

## Abstract

Statistical evaluation of empirical data is the basis of the modern scientific method. Available tools include various hypothesis tests for specific data structures, as well as methods that are used to quantify the uncertainty of an obtained result. Statistics are pivotal, but many misconceptions arise due to their complexity and difficult-to-acquire mathematical background. Even though most studies rely on a frequentist interpretation of statistical readouts, the application of Bayesian statistics has increased due to the availability of easy-to-use software suites and an increased outreach favouring this topic in the scientific community. Bayesian statistics take our prior knowledge together with the obtained data to express a degree of belief how likely a certain event is. Bayes factor hypothesis testing (BFHT) provides a straightforward method to evaluate multiple hypotheses at the same time and provides evidence that favors the null hypothesis or alternative hypothesis. In the present perspective, we show the merits of BFHT for three different use cases, including a clinical trial, basic research as well as a single case study. Here we show that Bayesian statistics is a viable addition of a scientist's statistical toolset, which can help to interpret data.

## Introduction

The research community in biological, psychological, and medical fields has become increasingly introspective regarding our applied statistics. Common statistical techniques, such as null hypothesis significance testing using *p*-values, have been re-examined (1–3). Additional criticism concerns the ritualization of statistical analysis whereby researchers propose null hypotheses without alternative hypotheses, apply the 5% significance level to reject their null hypothesis and accept the unspecific alternative hypothesis to then continue repeating this procedure indefinitely (4). This highlights a frequent, detrimental misinterpretation: that a *p*-value below five percent (*p* < 0.05) suggests a “significant” effect. Statistical significance is not to be confused with clinical relevance [see (1) for theoretical discussions, and our previous work (5) for a demonstration within ALSALS/MND research]. For some investigators, the implied inference is that because they have successfully rejected the null hypothesis, they may now accept the alternative hypothesis, concluding that there is an effect (4, 6). Consequently, the American Statistical Association (ASA) clarified the appropriate usage of *p*-values (7): *p*-values on their own do not measure the probability of the null hypothesis being true or the probability that the data were produced by random chance, they do not measure the size of the proposed effect and they do not provide a good estimation of evidence regarding a model or hypothesis. Since then, several solutions have been proposed: applying the *p*-value correctly (8), redefining statistical significance to *p* < 0.005 for exploratory studies (9) and supplementing every *p*-value with Bayesian analysis (10, 11). These approaches are not mutually exclusive, can point in the same evidential direction and may be combined to maximize the information we derive from our data (12, 13).

Other problems range from inappropriate data transformations, incorrect use of statistical methods (14) to misinterpretation of statistical readouts (15, 16). For example, basic research often erroneously applies Student's *t*-test to categorical frequency data; in the spirit of self-correcting science, we point to our own previous work (17–19). Conclusions drawn from these erroneously conducted analyses are submitted, where often, they meet peer-reviewers who misguidedly do not insist on correction. The resultant manuscripts are later published to audiences who are themselves less critical with the conducted procedures because they rely on the established peer review processes, leading to a vicious cycle of publishing these bad practices and solidifying the current state. These issues are non-specific; they apply across the sciences. They may be remedied by improving our statistical education, as well as the peer reviewer and publication processes. Bayesian statistics—as outlined above—may contribute to improving our statistical conclusions. It is our aim to introduce Bayesian approaches for the amyotrophic lateral sclerosis research community, to help us improve our conclusions. First, we will briefly outline how they have been applied to this in the past, before demonstrating on our own data.

The Bayesian approach to probability was recognized as a valuable tool to maximize the efficiency of clinical trials back in 2010 (20). Since then, its applications have remained somewhat niche: in January 2021, PubMed hosted 18,171 publications under the MeSH major topic “amyotrophic lateral sclerosis”—only 40 of which applied Bayesian modeling techniques (0.002%). In 2013, Sreedharan and Brown (21) had outlined the importance of epistatic interactions between genetic variants of ALS/MND and epidemiological studies of environmental risk factors for the then-coming years of ALS/MND research. Colak, Kim (22) have since developed a joint Bayesian analysis, which explored phenotypic heterogeneity and epistasis. Prior Bayesian work in this area includes an epistasis mapping algorithm (BEAM), which outperformed previous epistasis mapping tools (23). Epidemiologically speaking, Bayesian parameter estimation has been applied to document the increasing prevalence of ALS/MND in Portugal (24) and to discover spatial clusters of ALS/MND (25). Dynamic Bayesian networks have been used to assess disease progression regarding communication, movement, swallowing, breathing and weight loss to high degrees of accuracy (26). This network specifically modeled the progression over time, instead of time-to-event modeling with common techniques (26). Bayesian networks have been shown to predict ALS/MND more accurately than other machine learning techniques (27). Throughout, Bayesian modeling techniques have been lauded as advantageous by the ALS/MND researchers who applied them.

These niche but important Bayesian contributions to our field necessitate an introduction to Bayesian thinking and modeling techniques for the wider ALS/MND research community—which is what we aim to provide here. To appeal to a broad range of researchers, we will draw on three facets of ALS/MND research: clinical trials, basic research, and single cases. As such, this perspective offers applied methodologies for evidence quantification and future directions for statistical analysis.

## Introducing Bayes

Bayesian inference is named after the eighteenth century Presbyterian minister Thomas Bayes. His theorem describes an event's probability, after the occurrence of a different independent prior event (see Equation 1).


$$\begin{array}{l} P\left(M|data\right)\;=\;\frac{P\left(data|M\right)P\left(M\right)}{P\left(data\right)} \end{array}$$

The event in question is our hypothesis model (M). Prior to data analysis, researchers specify a *prior probability [P(M)]* of the hypothesis being supported by their data, once they have been collected (see Table 1). As a naïve guess, all possible hypotheses (including the null hypothesis) can have the same probability, for example, when there is no prior knowledge about the experimental outcome. P(data|M) is readily available because these hypotheses have a distributional assumption and therefore the likelihood of the data can be calculated. Researchers then collect and analyse their data [P(data)] to update the prior probability. This yields the *posterior probability [P(M|data)]*. This *posterior probability* can be interpreted as the probability of the hypothesis after the occurrence of the data. After this observation, our belief in the hypothesis can be strengthened or weakened. Of the numerous statistical approaches derived from Bayesian probability, we will focus on *Bayes factor hypothesis testing (BFHT)* which draws on the *Bayes factor (BF)* as a measure of evidential strength (13, 28). Commonly used measures in BFHT inference are listed in Table 1. The BF measures the relative plausibility of the competing hypotheses—null vs. alternative hypothesis—after the data have been analyzed (13). It can be calculated in favor of the null model (BF_01_), or the alternative model (BF_10_) by dividing their posterior probabilities [P(M|data)]. To obtain the evidence favoring the null hypothesis H_0_, divide the posterior probability of H_0_ by the posterior probability of the alternative hypothesis H_1_ to get BF_01_. To obtain the evidence in favor of H_1_ (i.e., BF_10_), swap denominator and numerator. This way, BFHT offers direct, probabilistic evaluation of several hypotheses representing different possible effects, facilitating a conclusion regarding which of them is most probable, and how much more probable it is compared to the others (1, 3, 6, 9, 28, 29). The conclusions provided by BFHT fall on a continuum between supporting the null hypothesis, being inconclusive, or supporting an alternative hypothesis (30). However, while *p*-values are interpreted utilizing a chosen cut-off (e.g., *p* < 0.05, *p* < 0.01, *p* < 0.001), BF can be interpreted without them. For example, when BF_10_ = 30, we can conclude “H_1_ is 30 times as likely as H_0_, according to my data.” This is intuitive without a cut-off. It is up to the researchers to make informed decisions as to which cut-off they consider sufficiently convincing. Additionally, qualitative descriptions of evidential strength may be applied to BF (e.g., BF < 3 and > 1/3 “anecdotal,” 3 < BF < 10 or 1/3 > BF > 1/10 “moderate,” BF > 10 or BF < 1/10 “strong”) (30).

**Table 1:** Commonly reported statistics in Bayesian inference.

**Notation/**	**Full name**	**Interpretation**
**abbreviation**		
Prior	Prior distribution	Distribution of the effect size, as assumed prior to data collection/analysis
Posterior	Posterior distribution	Actual distribution of the effect size after the data at hand have been analyzed
P(M)	Prior model probability	Probability of this particular statistical model being supported by the data at hand, as assumed prior to data collection/analysis
P(M|data)	Posterior model probability	Posterior probability of this particular model being supported by the data at hand, after they have been analyzed
BF	Bayes factor	The strength of evidence in favor of a given statistical model, relative to another statistical model (see below)
BF_01_	Bayes factor 0/1	The strength of evidence in favor of model 0, relative to model 1
BF_10_	Bayes factor 1/0	The strength of evidence in favor of model 1, relative to model 0
Error%	Stability of the BF	The range of the BF over the chosen Markov chain Monte Carlo iterations, e.g., BF_10_ = 10 with error% = 20 means that the BF_10_ ranged from 8-12

This framework thus quantifies evidence for the most probable hypothesis, instead of supporting the rejection of the null hypothesis. Being able to conclude “this effect is most probable according to the data” is more informative than “the absence of any effect is unlikely, given the data and hypothetically existing, more extreme data.” Once an effect has been deemed sufficiently probable, we may estimate its plausible values using *credible intervals (CI)*. These condition on the known data, providing 95% certainty that the estimated parameter's true value—say, an effect size—lies within their bounds (28). This differs from frequentist confidence intervals which merely contain the true effect in a fixed number of samples, see Morey et al. (31).

## Applied Examples of Bayesian Inference

### Bayesian Hypothesis Testing and Parameter Estimation in Clinical Trials

**Figure 1:**
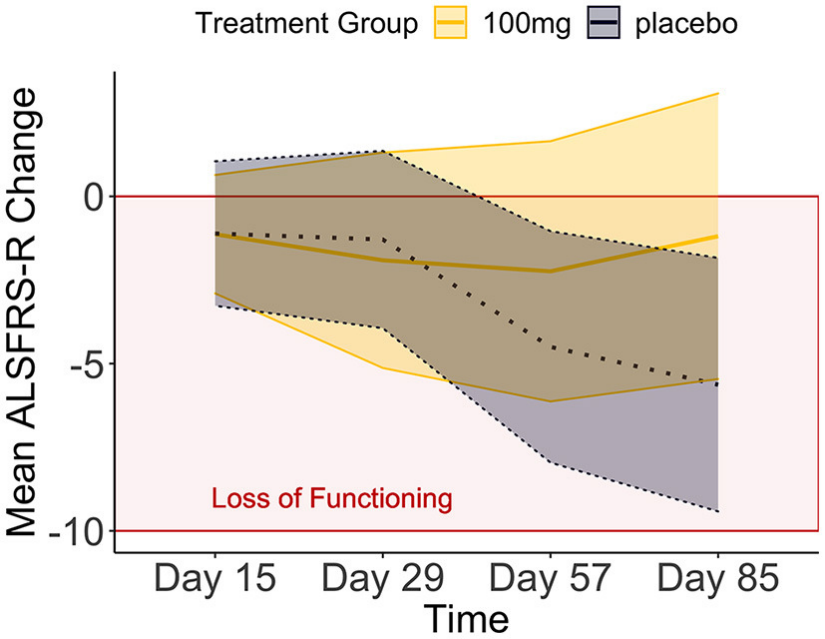
The means and credible intervals of motor progression over the tofersen phase II trial (synthetic data).

**Figure 2:**
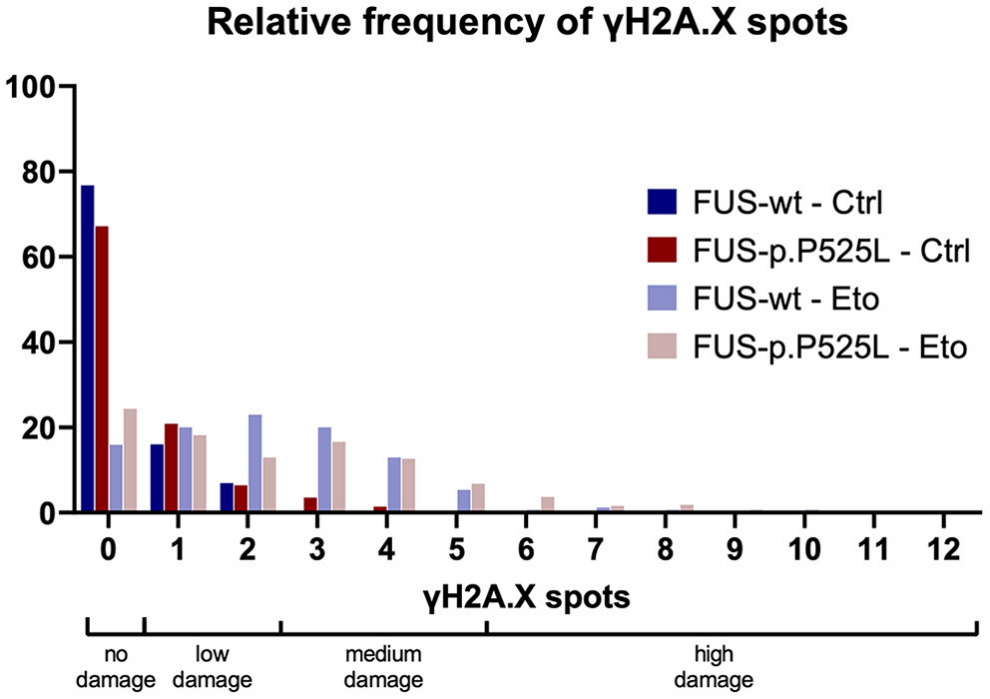
The relative frequency of γH2A.X spots (previously published data).

We applied Bayesian hypothesis testing to a synthetic data set based on Biogen's phase I/II trial of tofersen (32). The code to generate the synthetic tofersen dataset, the dataset itself as JASP file and PDF-based results can be obtained from the Open Science Framework, at https://osf.io/6cpf9/. We re-created the motor progression of the placebo and 100 mg tofersen treatment groups from the mean and confidence intervals published in Supplementary Table 3 of (32). The confidence intervals allowed us to derive standard deviations assuming a t-distribution for small samples (33). The outcome of motor progression was measured by the mean change of the revised amyotrophic lateral sclerosis functional rating scale [ALSFRS-R, (34)]. We used two independent variables, between-subjects treatment group (placebo vs. 100 mg tofersen) and within-subjects day (15th, 29th, 57th, 85th day of the study). Comparing our Figures 1, 2 of (32), we see that the re-creation was successful as our progression mirrors the original data's.

Three hypotheses were of interest (Table 2):

1) The null model, representing the absence of effects in our data,
2) The treatment effect model, representing the expectation that 100 mg of tofersen will slow down ALSFRS-R progression (“Treatment Group” in Table 2),
3) The interaction between treatment group and time, representing the hypothesis that the treatment groups will progress differently over time in their ALSFRS-R.

**Table 2:** Model comparison for the clinical trial data.

**Models**	**P(M)**	**P(M|data)**	**BF_**M**_**	**BF_**10**_**	**error %**
Null model (incl. subject)	0.2	0.37	2.38	1.00	
Time	0.2	0.23	1.22	0.63	0.45
Treatment Group	0.2	0.19	0.92	0.50	0.8
Time + Treatment Group	0.2	0.12	0.56	0.33	3.69
Time + Treatment Group +	0.2	0.08	0.36	0.22	0.95
Time * Treatment Group					

*All models include subject*.

We compared these hypotheses using a mixed measures analysis of variance (ANOVA) in *Jeffreys's Amazing Statistics Program* (JASP, 35). JASP was set to report the null model on top of the comparison table (Table 2) and provide the BF_10_ in favor of the alternative models listed below. In addition to the listed models, the sole main effect of time and the combined, independent main effects of time and treatment group were included in the model comparison. A priori, we assumed all hypotheses to be equally probable: P(M) = 0.20. Mean loss of functioning over time and by treatment group are depicted in Figure 1, surrounded by 95% credible intervals. These 95% credible intervals provide us 95% certainty, that the loss of functioning falls within the depicted bounds (Figure 1). Note that the smaller width of the credible intervals on days 15 and 29 reflects less uncertainty about the mean loss of functioning than the greater intervals on days 57 and 85. Frequentist confidence intervals do not facilitate inferences about the plausibility of the estimated mean loss of functioning, they do not contain reasonable values mean loss of functioning may take, and they do not indicate precision of the estimates (31). Following data analysis, the null model's posterior probability increased to P(M|data) = 0.373, meaning that our belief in the null model strengthened from 20 to 37.3%. Contrastingly, our belief in the treatment effect decreased from 20 to 18.7% (P(M|data) = 0.187). From these posterior probabilities [P(M|data)], we derived the BF_10_ by dividing P(M|data) of H_1_ by P(M|data) of H_0_: 0.187/0.373 = 0.502. At BF_10_ = 0.502, our data are half as probable under the treatment effect hypothesis compared to the null hypothesis, which corresponds to BF_01_ = 1.99 if we swap denominator and numerator. BFHT informed us that our data are twice as likely under the null hypothesis compared to the treatment effect hypothesis. By comparison, a frequentist ANOVA would inform us that the probability of our synthetic data—or hypothetical, more extreme data—occurring would be 14%, if the null hypothesis were true [*p* = 0.140, F_(3, 60)_ = 1.88]. We consider the former conclusion more informative regarding the treatment effect. The treatment effect explained 29% of the variance in motor progression (R^2^ = 0.29, 95%CI [0.14|0.43].

Our data reduced our belief in the hypothesis that treatment groups will progress differently over time from 20 to 8.2% [P(M|data) = 0.082]. With a BF_10_ = 0.22 and conversely, BF_01_ = 4.54, the null hypothesis is 4.54 times more probable than the interaction model. This interaction model explained 35% of the variance in motor progression (R^2^ = 0.35, 95%CI [0.21|0.47]).

We estimated the effect size using Kendall's tau correlation coefficient (τ_b_). As an interaction between Time^*^Treatment Group was improbable (BF_01_ = 4.54, favoring H_0_ over the interaction in Table 2), we correlated treatment group and progression speed of day 85. We observed a large effect size of τ = −0.312, which with a probability of 95%, fell between −0.543 to −0.003. This indicates major uncertainty about tofersen's effect size as the credible interval indicates that nearly a quarter of all possible values (τ_b_ range from −1 to +1) are deemed plausible.

In conclusion, we cannot accept or reject the treatment effect based on the mixed-measures ANOVA in Table 2 (BF_10_ = 0.502, in favor of the treatment effect) because the evidence in these data is insufficient. This sufficiency of evidence is determined by the BF_10_: concluding that the treatment effect is 0.5 times better than its absence is unconvincing either way. However, if the effect does exist, it ranges from very small to very large (95%CI between −0.003 and −0.543). This range is consistent with inconclusive BF as some individuals may exhibit a large treatment effect, and some may exhibit no treatment effect. Supplementary Table 1 compares the conclusions possible based on both statistical frameworks. Clinically, our synthetic data inform us that we cannot be certain if tofersen works—but if it works, any effect varies considerably between patients. This is congruent with tofersen's phase III results, analyzed and published using frequentist probability after we first submitted our manuscript.

### Bayesian Hypothesis Testing for Categorical Data in Basic Research

Biomedical basic research aims to understand molecular mechanisms underlying diseases as well as to develop disease-modifying treatments and their translation to the clinics. Therefore, reliable inference from data is pivotal, as it leads to more sophisticated experimental approaches or eventually to clinical trials.

We wanted to examine whether a Bayesian approach is helpful in biomedical basic research and re-evaluated a subset of previously published own data (19). We quantified the amount of DNA-damage in a FUS-ALS cell culture model, γH2A.X, a marker for double strand breaks (DSBs), was stained with immunofluorescence. We compared a wild type FUS cell line with its isogenic control carrying the ALS-causing p.P525L mutation. The cells were investigated under control condition (Ctrl) as well as after 1 hour treatment with 5 μM of the TOP2 inhibitor Etoposide (Eto), which is inducing DSBs. The number of γH2A.X-positive foci per nucleus was then counted and evaluated. We conducted four independent biological replicates, took at least five pictures, and evaluated at least 47 nuclei per condition. Count of γH2A.X-positive foci per nucleus present mutually exclusive categorical data—not continuous data. To represent a frequentist approach, we used a chi-square-test, instead of the *t*-test previously employed in our publication. We categorized the raw data into groups of “no damage” (0 spots), “low damage” (1–2 spots—reflecting the wild type FUS cell line under Ctrl conditions), “medium damage” (3–5 spots—reflecting the wild type FUS cell line with Eto treatment) and “high damage” (>5 spots), see Figure 2. The contingency table was analyzed and the following statistical parameters were calculated: [Ctrl: χ^2^(3, *N* = 601) = 18.115, *p* < 0.001, Cramer's V = 0.174; Eto: χ^2^(3, *N* = 495) = 13.712, *p* = 0.003, Cramer's V = 0.166]. These results indicate that we may reject the null hypothesis that there is no association, and that any potential association would be weak. For the Bayesian approach we examined the contingency table and assumed a Poisson distribution of the data because we sampled without restriction on either the cells analyzed or DSBs. This analysis suggested not only to reject the null hypothesis but, furthermore, provided strong evidence of an association between the genotype and the amount of DNA-damage. In our data, the presence of the association was 11 times more likely than its absence under control conditions, and 13 times more likely under Eto treatment (Ctrl: BF_10_ = 11.304, Eto: BF_10_ = 13.711, Supplementary Table 2).

The Bayesian approach for evaluation of this data set led to a similar conclusion as the frequentist approach, however, with the important difference that instead of merely rejecting the null hypothesis without specification of a distinct alternative hypothesis in the chi square test, the Bayesian approach favored the hypothesis that there is an association of the genotype with the amount of DSB.

### Bayesian Inferences in Single Case Studies

Historically, single case studies have contributed meaningfully to ALS-FTSD research: they have been used to describe behavioral disturbances (35), co-occurrence between ALS/MND and other diseases (36, 37), verb processing deficits (38), longitudinal observations (39, 40), and rare familial forms (39, 41–43). Single cases investigate whether the index patient is qualitatively and quantifiably different from a healthy control population (44).

Here, the Bayesian approach facilitates probabilistic conclusions about the index patient (44–46). This enables comparisons of neuropsychological measures across different scales while providing detailed clinical information about cognitive profiles by capturing the range of uncertainty using CI. Frequentist techniques treat the obtained dependent variables (or outcomes of interest)—as fixed but unknown whereas Bayesian techniques treat them as random variables which have probability distributions, meaning an effect's size can be estimated using that probability distribution (44). With frequentist techniques, an effect size cannot be calculated because the data from the index patient is treated as a fixed variable and the distribution of the control cohort is compared to it. This cohort would either be below the threshold of interest (the index patient)—or not, depriving us of probabilistic conclusions about the index patient's abnormality.

We illustrate these benefits using the *SingleBayes_ES.exe* program from our previous work, see Supplementary Table 3 in Temp, Dyrba (39). To summarize briefly: this program's modeling is based on a prior distribution whose unknown mean and unknown variance are obtained by an observation from a standard normal distribution and a random value from a chi-square distribution on n-1*df*, respectively. The point estimate of abnormality is calculated on the condition that these are the true mean and variance, and the process is reiterated 100,000 times. Further details can be obtained in the description of Experiment 1 by Crawford and Garthwaite (44). Table 3 lists the cognitive assessment, the number of healthy controls, their mean and standard deviation (SD), the male index patient's performance, a *p*-value based on a two-tailed Bayesian hypothesis test, the estimated percentage of controls who scored lower than the patient including 95% CI (point estimate of abnormality), and the estimated effect size including 95% CI (39).

For the digit span forward task (Table 3), the probability that a control might obtain a score below the index patient was 7% (*p* = 0.070), and the estimated percentage of healthy controls who might obtain a score below 6 was 3.49%, with a 95% probability that the true percentage fell between 0.24 and 12.41%. In a frequentist context, the interpretation of the interval estimates would be: “if we could compute a confidence interval for a large number of control samples collected in the same way as the present control sample, about 95% of them would contain the true percentage of the population with scores lower than [six]” (44). As Bayesians, we estimate the proportion of controls whose digit span forward performance fell below that of the index patient which gives us the probability that any control might score similarly to the index patient. Thus, the Bayesian approach provides more conclusive evidence for the hypothesis that the index patient deviates from the control sample.

Commonly, an index patient's scores are evaluated with z scores under a normal distribution, treating the statistics from the normative samples as parameters. This method frequently exaggerates the abnormality of the index patient, especially with a small normative sample (for mathematical simulations on this, see 45). The Bayesian method presented here treats the control sample statistics as such, minimizing this bias. The index patient (Table 3) fell two standard deviations below control performance, with a 95% probability that this effect sizes falls between −2.82 and −1.16. Consequently, the Bayesian method supplements the above evidence with information on how strongly the index patient deviates from the control sample without exaggerating the abnormality.

Bayesian single case analyses are available for comparisons of a single index patient to a control sample without covariates (44, 45) and with covariates (46), and for comparing two single cases to one another (47). A catalogue of free statistical software has been compiled by John Crawford, and can be accessed here. These programs operate only in Windows but the R package “singcar” has recently been developed, so non-Windows users can implement these techniques (48). Notably, while these methods were developed for cognitive tests, they can and have been applied to other measures, such as psychiatric well-being (49), hippocampal volume (50), and positron emission tomography (51), making them informative for single case researchers of any discipline.

## Conclusions

Our perspective highlights the benefits by giving examples of BFHT in clinical trials and basic research, as well as parameter estimation in single case studies. While frequentist approaches may hinder inference by failing to test the alternative hypothesis, we can distinguish between evidence supporting the alternative hypothesis (e.g., our cell culture data), evidence supporting the null hypothesis and the absence of conclusive evidence (e.g., our synthetic tofersen data) using Bayes factors. Once evidence for a model is established, we can use CI to further quantify our certainty about a parameter's size. CI interpretation is much more straightforward than interpretation of confidence intervals, which provide only prospective estimation of not yet obtained data, while CIs are derived from already obtained data and can be used to characterize them.

Translation of basic research into useful treatments is an important, however, often unsuccessful endeavor. This is partly the case due to limitations of the disease models used. Modeling these diseases *in vitro* within a reasonable time frame and using functional assays with readily obtainable readouts for drug screening (e.g., cell survival, neuronal activity, energy consumption) imposes the challenge of detecting and interpreting small effect sizes. Some of the variance can be controlled by minute experimental setup, while other factors like biological variances between replicates, lot-to-lot differences of substances or technical replicates are—albeit well-documented—usually not considered in statistical procedures to simplify the model and allow easier interpretation of the results. However, they still add variance to the data and therefore may mask small effects. BFHT allows a direct comparison of multiple alternative hypotheses and an analysis of effects of all variables across all evaluated alternative hypotheses. Similar comparisons are possible with likelihood ratio tests or various information criteria which may also be applied in frequentist contexts and have been applied in around 25% of ALS-related literature in PubMed. BFHT offers a straightforward way to compare these confounding variables with meaningful ones and subsequently adding them to the null model and keeping their effect included across all compared models. Addition of confounding variables to the null model reduces noise and enables the scientist to observe smaller effects of the meaningful variables like genotypes and treatments.

Similarly, Bayesian probability allows us to discriminate between groups of patients as well as an individual patient and a control group. It can be used alongside frequentist approaches and provide a framework to include prior information and harness more information from data. Combined, these approaches can improve our clinical trials, diagnostics, and phenotyping, as well as our scientific conclusions, while avoiding failure within clinical translation, e.g., due to disregarding a proper testing of the alternative hypothesis in basic science.

Regardless, Bayesian inference is not a one-size-fits-all solution to all our statistical problems; it is still susceptible to model misspecification due to violated assumptions [e.g., normality, heterogeneity (52)], and it is equally vulnerable to unintentional (or intentional) misuse. Aspiring and already-practicing Bayesians should also be aware of on-going, fundamental discussions and developments in the field; non-exhaustive examples include critiques aimed at the application of Bayes factors in ANOVA designs with continuous outcomes (53) and how to accordingly modify Bayes factors for various test designs (54–56), and deliberations on how to choose the most suitable prior distribution [see, for example, (57, 58)].

## Further Reading

We would like to pinpoint our readers to seminal textbooks introducing Bayesian statistics (59–62). Readers who prefer articles instead are wellserved by recent publications illuminating the theoretical background (3, 6, 63). Those looking for information about Bayesian “translations” of common statistical techniques may find them as listed: *t*-tests (64, 65), ANOVA (30, 52, 66) and correlations (30, 67), with further details on clinical trials (68–74). Commercial software packages such as SPSS, STATA or SAS now include Bayesian approaches. Those without access to commercial software can turn to JASP (75), which also offers a Bayesian port for jamovi; and a multitude of packages in R (76), Research applying Bayesian statistics has increased steadily over the past three decades (77). In 1975, Lindley prophesied that the twenty-first century would have become Bayesian by 2020—let's make it so (78).

## Data Availability Statement

The datasets presented in this study can be found in online repositories. The names of the repository/repositories and accession number(s) can be found in the article/Supplementary Material.

## Ethics Statement

The synthetic clinical trial did not involve any human participants as it was generated using R code. The basic research dataset was generated in a study approved by the Ethical Committee of the Technische Universität Dresden (EK45022009, EK393122012) and by the Ethical Committee of the University of Ulm (Nr. 0148/ 2009) and patients and controls gave their written consent prior to skin biopsy. The cited single case research project was approved by the Medical Ethics Committee of the University of Rostock (A2010-32 and A2011-56).

## Author Contributions

AGMT conceptualised the manuscript, generated and analysed the synthetic tofersen data, and wrote the original draft of the following sections: the overall introduction, Introducing Bayes, Bayesian Hypothesis Testing and Parameter Estimation in Clinical Trials, Bayesian Inferences in Single Case Studies, Conclusions and Further Reading. AGMT is also the first author of the paper on which the section bayesian inferences in single case studies is based. MN collected and published the dataset described in the Basic Research section and provided feedback on the final draft. AH acquired the funding for this manuscript, supervised MND's project, contributed to the conceptualisation of the Basic Research section, and provided feedback on the final draft. HG conceptualised the Basic Research section, conducted the analyses therein, and wrote the Basic Research section and contributed majorly to the remainder of manuscript. All authors contributed to the article and approved the submitted version.

## Funding

This work was supported in part by the Boris Canessa ALS/MND foundation and the professional cyclist André Greipel and his Fight ALS/MND initiative supporting the Cognition in ALS/MND working group at DZNE Rostock. AH is supported by the Hermann und Lilly Schilling-Stiftung für medizinische Forschung im Stifterverband.

## Conflict of Interest

The authors declare that the research was conducted in the absence of any commercial or financial relationships that could be construed as a potential conflict of interest.

## Publisher's Note

All claims expressed in this article are solely those of the authors and do not necessarily represent those of their affiliated organizations, or those of the publisher, the editors and the reviewers. Any product that may be evaluated in this article, or claim that may be made by its manufacturer, is not guaranteed or endorsed by the publisher.
