# Supplementary material for: Applied Bayesian Approaches for Research in Motor Neuron Disease
Source: Front Neurol. 2022 Mar 24;13:796777. doi: 10.3389/fneur.2022.796777 (PMC8987707; doi:10.3389/fneur.2022.796777)
Supplement: Supplementary file 1 [file Table_1.docx]

| Supplementary Table 1. Comparison between the conclusions facilitated by classical NHST and Bayesian hypothesis testing. | | | | | | |
| --- | --- | --- | --- | --- | --- | --- |
|  | Null hypothesis significance testing | | | Bayes factor hypothesis testing | | |
| Model | p-value | ω^2^ | Conclusion | BF_01_ | BF_10_ | Conclusion |
| Day | .142 | .022 | We have failed to reject the null hypothesis because there is only a 14.2% probability of the observed result, or more extreme results, if the null hypothesis were true. Consequently, no support for the null hypothesis, or any other hypothesis, can be established. | 1.16 | 0.628 | The null hypothesis is 1.16 times more probable than the main effect of Day, according to our data. Consequently, no conclusive evidence for either hypothesis can be established. |
| Group | .298 | .003 | We have failed to reject the null hypothesis because there is only a 29.8% probability of the observed result, or more extreme results, if the null hypothesis were true. Consequently, no support for the null hypothesis, or any other hypothesis, can be established. | 1.99 | 0.502 | The null hypothesis is two times more probable than the main effect of Group, according to our data. Consequently, no conclusive evidence for either hypothesis can be established. |
| Day*Group | .162 | .019 | We have failed to reject the null hypothesis because there is only a 16.2% probability of the observed result, or more extreme results, if the null hypothesis were true. Consequently, no support for the null hypothesis, or any other hypothesis, can be established. | 4.54 | 0.220 | The null hypothesis is 4.5 times more probable than the interaction between Day and Group. Consequently, the null hypothesis is supported over the interaction hypothesis. |

| Supplementary Table 2. Comparison of conclusions by different classical NHST-methods and Bayesian hypothesis testing. | | | | | | |
| --- | --- | --- | --- | --- | --- | --- |
|  | Null hypothesis significance testing | | | Bayes factor hypothesis testing | | |
| Method | p-value | Effect size | Conclusion | BF_01_ | BF_10_ | Conclusion |
| Chi-square- test (Ctrl) | <.001 | .174 (Cramer’s V) | We were able to reject the null hypothesis, because there is less than 0.1% probability of the observed result, or more extreme results, if the null hypothesis were true. Consequently, no support for the null hypothesis can be established, and there is a weak association. | 11.30 | 0.08 | An association of the genotype with DNA-damage is 11.3 times more probable than no association. Consequently, the alternative hypothesis is strongly supported over the null hypothesis. |
| Chi-square- test (Eto) | .003 | .166 (Cramer’s V) | We were able to reject the null hypothesis, because there is 0.3% probability of the observed result, or more extreme results, if the null hypothesis were true. Consequently, no support for the null hypothesis can be established, and there is a weak association. | 13.71 | 0.07 | An association of the genotype with DNA-damage is 11.3 times more probable than no association. Consequently, the alternative hypothesis is strongly supported over the null hypothesis. |

| Supplementary Table 3. Case study table, adapted from Temp et al. (2021). | | | | | | | | | | | | | | |
| --- | --- | --- | --- | --- | --- | --- | --- | --- | --- | --- | --- | --- | --- | --- |
| Assessment | Healthy HC | | | | The patient | | Two-tailed Bayesian Hypothesis Test |  | Estimated percentage of the HC obtaining a lower score than the patient | |  | Estimated effect size (z_HC_) | |  |
|  | N | Mean | | SD | |  | Probability |  | Point | 95%CI |  | Point | 95%CI |  |
| MoCa | 17 | 29 | 2 | | 29 | | .999 |  | 50.00 | 31.74 to 68.29 |  | 0.00 | -0.48 to 0.48 |  |
| Digit Span fw.^†^ | 17 | 8 | 1 | | 6 | | .070 |  | 3.49 | 0.24 to 12.41 |  | -2.00 | -2.82 to -1.16 |  |
| Digit Span bw. ^†**^ | 17 | 7 | 1 | | 2 | | <.001 |  | 0.01 | 0.00 to 0.07 |  | -5.00 | -6.77 to -3.21 |  |
| Learning | 17 | 51 | 7 | | 52 | | .891 |  | 55.44 | 36.80 to 73.20 |  | 0.14 | -0.34 to 0.62 |  |
| Recall 1 | 17 | 11 | 3 | | 12 | | .750 |  | 62.50 | 43.63 to 79.32 |  | 0.33 | -0.16 to 0.82 |  |
| Recall 2 | 17 | 11 | 3 | | 11 | | .999 |  | 50.01 | 31.74 to 68.29 |  | 0.00 | --0.48 to 0.48 |  |
| Recognition | 17 | 12 | 3 | | 12 | | .999 |  | 50.01 | 31.74 to 68.29 |  | 0.00 | -0.48 to 0.48 |  |
| phon. VF (Σ) | 17 | 15 | 3 | | 21 | | .070 |  | 96.51 | 87.57 to 99.76 |  | 2.00 | 1.15 to 2.82 |  |
| phon. VF (Index) | 17 | 3.4 | 0.84 | | 2.19 | | .180 |  | 9.04 | 1.72 to 22.88 |  | -1.44 | -2.11 to -0.74 |  |
| sem. VF (Σ) | 17 | 21 | 5 | | 27 | | .261 |  | 86.97 | 71.21 to 96.54 |  | 1.20 | 0.56 to 1.82 |  |
| sem. VF (Index) | 17 | 2.21 | 0.62 | | 2.19 | | .976 |  | 48.78 | 30.61 to 67.15 |  | -0.03 | -0.51 to 0.44 |  |
| TMT B/A^†*^ | 17 | 2.31 | 0.61 | | 3.79 | | .031 |  | 98.43 | 92.74 to 99.96 |  | -2.43 | 1.46 to 3.37 |  |
| ToL Correct | 17 | 4 | 1 | | 4 | | .999 |  | 50.01 | 31.74 to 68.29 |  | 0.00 | -0.48 to 0.48 |  |
| ToL Errors | 17 | 0 | 1 | | 0 | | .999 |  | 50.01 | 31.74 to 68.29 |  | 0.00 | -0.48 to 0.48 |  |
| ToL Moves | 17 | 37 | 4 | | 37 | | .999 |  | 50.01 | 31.74 to 68.29 |  | 0.00 | -0.48 to 0.48 |  |
